# Authors reply regarding “A‐V‐V‐A response to single atrial premature depolarization in a narrow QRS tachycardia: What is the mechanism?”

**DOI:** 10.1002/joa3.13107

**Published:** 2024-06-24

**Authors:** Shingo Yoshimura, Yosuke Nakatani, Kenichi Kaseno, Kohki Nakamura, Shigeto Naito

**Affiliations:** ^1^ Division of Cardiology Gunma Prefectural Cardiovascular Center Maebashi Gunma Japan

## Abstract

We respond to a letter by Dr. A. Goyal. If the tachycardia were junctional ectopic tachycardia (JET), the occurrence of the ventriculoatrial block following an atrial premature depolarization could not be explained. Therefore, we conclude that atrioventricular nodal reentrant tachycardia was more likely than JET.

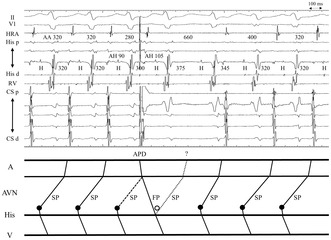

We read the letter by Dr. A. Goyal^1^ with great interest and sincerely appreciate the profound insights Dr. Goyal provided regarding the case report we published.^2^ We used the response to a single atrial premature depolarization (APD) delivered during tachycardia to differentiate junctional ectopic tachycardia (JET) from atrioventricular nodal reentrant tachycardia (AVNRT). As Dr. Goyal pointed out, this response applies to tachycardias with a short ventriculoatrial interval (from the ventricle to the high‐right atrium [V‐HRA]) but not to tachycardias with a long V‐HRA interval because perturbation of the next tachycardia beat confirms AVNRT only when APD does not affect the immediate beat. We agree with these arguments but conclude that AVNRT was more likely than JET based on the following explanation.

In the described case, the ventriculoatrial (VA) block occurred following an APD.^2^ A long HA interval during tachycardia suggests that retrograde conduction occurs through the slow pathway (SP), regardless of whether tachycardia were AVNRT or JET. If tachycardia were a JET, the wavefront from the APD would propagate via anterograde fast pathway, activating the atrioventricular junction before the JET beat and advancing the immediate His‐bundle potential. We estimated the effective refractory period of the retrograde SP to be <250 ms. Therefore, as the retrograde SP will have a sufficient interval to recover from refractoriness after APD, the wavefront would consequently encroach on the retrograde SP and not cause a VA block. In contrast, the VA block can be explained by the conduction block in the upper common pathway in fast–slow AVNRT.

We considered other diagnostic findings also but none were useful. Although evaluating the ΔHA interval (HA interval during pacing minus HA interval during tachycardia) is useful for differentiating between typical AVNRT and JET,^3^ it does not apply to long RP tachycardia. Additionally, the response to atrial overdrive pacing during tachycardia was not helpful in this case because a double ventricular response could be possible.^4^

## FUNDING INFORMATION

N/A.

## CONFLICT OF INTEREST STATEMENT

The authors declare no conflicts of interest.

## INFORMED CONSENT

N/A.

## ETHICS STATEMENT

N/A.

## CLINICAL TRIAL REGISTRATION

N/A.

## References

[1] Goyal A . The letter to the editor regarding “A‐V‐V‐A response to single atrial premature depolarization in a narrow QRS tachycardia: what is the mechanism?”. J Arrhythm. 2024. 10.1002/joa3.13075 PMC10692860 38045453

[2] Yoshimura S , Nakatani Y , Kaseno K , Nakamura K , Naito S . A‐V‐V‐A response to single atrial premature depolarization in a narrow QRS tachycardia: what is the mechanism? J Arrhythm. 2023;39:965–968. doi:10.1002/joa3.12924 38045453 PMC10692860

[3] Srivathsan K , Gami AS , Barrett R , Monahan K , Packer DL , Asirvatham SJ . Differentiating atrioventricular nodal reentrant tachycardia from junctional tachycardia: novel application of the delta H‐A interval. J Cardiovasc Electrophysiol. 2008;19:1–6. 10.1111/j.1540-8167.2007.00961.x 17916156

[4] Fan R , Tardos JG , Almasry I , Barbera S , Rashba EJ , Iwai S . Novel use of atrial overdrive pacing to rapidly differentiate junctional tachycardia from atrioventricular nodal reentrant tachycardia. Heart Rhythm. 2011;8:840–844. doi:10.1016/j.hrthm.2011.01.011 21220046

